# Avertin®, but Not Volatile Anesthetics Addressing the Two-Pore Domain K+ Channel, TASK-1, Slows Down Cilia-Driven Particle Transport in the Mouse Trachea

**DOI:** 10.1371/journal.pone.0167919

**Published:** 2016-12-08

**Authors:** Ghulam Murtaza, Petra Mermer, Uwe Pfeil, Wolfgang Kummer

**Affiliations:** Institute of Anatomy and Cell Biology, Justus-Liebig-University and German Center for Lung Research (DZL), Excellence Cluster Cardio-Pulmonary System (ECCPS), Giessen, Germany; Bose Institute, INDIA

## Abstract

**Rationale:**

Volatile anesthetics inhibit mucociliary clearance in the airways. The two-pore domain K^+^ channel, TASK-1, represents one of their molecular targets in that they increase its open probability. Here, we determine whether particle transport speed (PTS) at the mucosal surface of the mouse trachea, an important factor of the cilia-driven mechanism in mucociliary clearance, is regulated by TASK-1.

**Methodology/Results:**

RT-PCR analysis revealed expression of TASK-1 mRNA in the manually dissected and laser-assisted microdissected tracheal epithelium of the mouse. Effects of anesthetics (isoflurane and Avertin®) and TASK-1 inhibitors (anandamide and A293) on ciliary activity were investigated by assessment of PTS at the mucosal surface of the explanted and opened murine trachea. Neither TASK-1 inhibitors nor isoflurane had any impact on basal and ATP-stimulated PTS. Avertin® reduced basal PTS, and ATP-stimulated PTS decreased in its presence in wild-type (WT) mice. Avertin®-induced decrease in basal PTS persisted in WT mice in the presence of TASK-1 inhibitors, and in two different strains of TASK-1 knockout mice.

**Conclusions/Significance:**

Our findings indicate that TASK-1 is expressed by the tracheal epithelium but is not critically involved in the regulation of tracheal PTS in mice. Avertin® reduces PTS independent of TASK-1.

## Introduction

Mucociliary clearance (MCC) is the defence mechanism by which airways get rid of inhaled pathogens and particles from environment and clean themselves [1, 2]. Life threatening complications are associated with impaired MCC from the airways [3]. Various anesthetics decrease overall MCC as measured by scintigraphy after inhalation of radiolabelled particles [4]. Yet, the underlying mechanisms are only poorly understood. The rate of MCC is determined by cilia-driven transport of the mucus layer, the amount and viscosity of the mucus, and, in case of the lower airways, by coughing, and all these mechanisms might be differentially affected via various intracellular signalling pathways.

The background potassium channels TASK-1 and TASK-3 (TWIK-related acid-sensitive K^+^-channel 1 and 3; official gene names: potassium two pore domain channel subfamily K members 3 and 9 = *kcnk3* and *kcnk9*, respectively) emerged as molecular target for volatile anesthetics such as halothane and isoflurane, as they directly activate these channels [5], and mice lacking TASK-1 show resistance to anesthesia [6]. TASK-1 is a two-pore domain K^+^ channel (K_2P_) with four transmembrane segments [7, 8]. In addition to a widespread distribution in the nervous system, it is expressed in ovine laryngeal mucosa [9] and in human non-small cell lung cancers [10]. Inhibition of TASK activity reduces background K^+^ influx leading to membrane depolarization. In carotid body glomus cells, it has directly been shown that this causes a rise in intracellular calcium concentration ([Ca^2+^]_i_) due to increased entry through voltage-gated Ca^2+^ channels [11], and this is also proposed as the underlying mechanism causing pulmonary artery smooth muscle cells constriction in response to the TASK inhibitor anandamide [12]. In ciliated cells of the respiratory epithelium, an increase in [Ca^2+^]_i_ causes a rapid increase in ciliary beat frequency (CBF) and lowering [Ca^2+^]_i_ is associated with a decrease in CBF [13, 14]. Hence, activation of these channels by anesthetics may ultimately lead to decrease in [Ca^2+^]_i_ and ciliary activity and thus resulting in reduced MCC. On the other hand, voltage-gated Ca^2+^ channels are not ubiquitously expressed in the respiratory epithelium and restricted to certain cell types and stages of development [15, 16], and coupling of membrane potential to [Ca^2+^]_i_ in ciliated cells might differ from that in glomus and smooth muscle cells. A study on mouse ciliated cells even argues for an increase in CBF in response to hyperpolarizing agents due to opening of non-voltage-gated Ca^2+^ channels [17]. In that case, anesthetics would even have a stimulatory effect on CBF if acting through TASK channels.

We addressed this issue by analyzing TASK-1 and -3 expression in the murine tracheal epithelium and measuring cilia-driven particle transport at the mucosal surface of the freshly explanted mouse trachea. In this mucus-free model, a wide range of regulators or chemicals can modulate CBF [18], and particle transport speed (PTS) is entirely dependent on ciliary activity but not on mucus composition [19]. In this model, we tested the effects of isoflurane, a TASK activator [20, 21], and Avertin® (tribromoethanol), an anesthetic with no known effect on TASK-1, along with those of TASK inhibitors (anandamide, A293). Two different strains of TASK-1 knockout (KO) mice [22, 23] were used to evaluate whether observed effects were mediated through this channel.

## Materials and Methods

### Animals

Animals of both sexes were used and maintained under standard laboratory conditions. Mice used were 14–16 weeks old and sacrificed by administration of an overdose of inhaled isoflurane in reverse transcription-polymerase chain reaction (RT-PCR) and by CO_2_ asphyxiation in PTS measuring experiments, respectively. All experiments were carried out according to the Guide for the Care and Use of Laboratory Animals published by the US National Institutes of Health and approved by the Regierungspräsidium Giessen, Germany (approval No. GI20/23, No. A7/2009). Mice used in our investigation were of the C57BL/6 background. One strain of TASK-1 KO mice described in detail previously [22] was a kind gift from Prof. W. Wisden, Imperial College London, and the other described previously [23] was provided through Prof. J. Daut, University of Marburg. KO mice from the colony were compared to age-matched wild-type (WT) mice housed in similar conditions. Genotyping of KO animals [22] was performed by PCR using genomic DNA obtained from either ear or tail samples with DNeasy Blood & Tissue Kit (Qiagen, Hilden, Germany) according to the manufacturer’s protocol. Red Taq (5 U/μl; Sigma, Taufkirchen, Germany) was used for PCR and PCR conditions were initial activation at 95°C for 3 min, 5 cycles with 95°C for 30 s, 60°C for 20 s, 72°C for 30 s, and finally 35 cycles with 95°C for 30 s, 57°C for 20 s, and 72°C for 30 s. Primers used were 5ˊ-TCATCGTGTGCAACCTTCACC-3ˊ, 5ˊ-CCTTCTATCGCCTTCTTGACG-3ˊ, 5ˊ-TGATGGCGAAGTAGAAGGAGC-3ˊ.

### RT-PCR

Expression of TASK-1 and TASK-3 transcripts in the heart, tracheal epithelium, trachealis muscle, and kidney (at least n = 3 for each) was determined by RT-PCR. The epithelium was collected by scrolling a cotton swab over the opened surface of the trachea. The trachealis muscle was dissected manually with laboratory scissors under visual control aided by a dissecting microscope. Total RNA was extracted using RNeasy Micro Kit (Qiagen, Hilden, Germany) according to the protocol provided by the manufacturer and was reverse-transcribed by applying the following conditions: Initially, 1 μg RNA present in 8 μl water was incubated with 1 μl 10x DNAse reaction buffer and 1 μl DNase (1 U/μl; Invitrogen, Darmstadt, Germany) for 15 min at 25°C to remove any genomic DNA contamination. Afterwards, samples were incubated with 1 μl ethylenediaminetetraacetic acid (25 mM) for 10 min at 65°C to inactivate the enzyme. Samples were then put on ice and RT mix [1 μl dNTPs (10 mM), 1 μl oligo-dT (50 μM), 4 μl 5x first strand buffer, 2 μl dithiothreitol (0.1 M), 1 μl super script II reverse transcriptase (RT, 200 U/μl; all reagents were purchased from Invitrogen except dNTPs which were from Qiagen)] was added. The cDNA samples were then PCR-amplified using TASK-1, TASK-3, and β-actin primers. The following conditions were used: 4 μl cDNA as template, 2 μl MgCl_2_ (25 mM), 2.5 μl 10x PCR buffer II, 0.5 μl of each primer (10 μM), 0.5 μl dNTPs (10 mM), 0.2 μl AmpliTaq Gold DNA Polymerase (5 U/μl; all reagents were obtained from Applied Biosystems, Darmstadt, Germany), and 14.8 μl H_2_O. The PCR was conducted using a thermal cycling profile of 95°C for 12 min, followed by 39 cycles of 20 s at 95°C, 20 s at 60°C, and 20 s at 72°C. β-Actin was used as housekeeping gene internal control. The cDNA of heart sample that was amplified successfully in the previous experiments was used as positive control to check that the PCR conditions could successfully amplify the target sequence. The absence of any contaminating genomic DNA was verified via the inclusion of reactions without adding RT during cDNA synthesis (ØRT). Samples were also processed without template (H_2_O). The primers used in RT-PCR were TASK-1 forward (Fw): 5ˊ-CCTTCTACTTCGCCATCACC-3ˊ, reverse (Rev): 5ˊ-GACACGAAACCGATGAGCAC-3ˊ, TASK-3 Fw: 5ˊ-CGCCCTCGAGTCGGACCATG-3ˊ, Rev: 5ˊ-ACCAGCGTCAGGGGGATACCC-3ˊ, GenBank accession No. NM_001033876, and β-actin Fw: 5ˊ-GTGGGAATGGGTCAGAAGG-3ˊ, Rev: 5ˊ-GGCATACAGGGACAGCACA-3ˊ, GenBank accession No. NM_007393. The amplicons were analyzed on 2% ethidium bromide-stained agarose gels, which were subjected to UV transillumination. Real-time RT-PCR was conducted as described earlier in [24]. All analyses were carried out in triplicate. The expression of TASK-1 as the gene of interest was normalized to β-actin as the housekeeping gene. The sequences of primer sets for TASK-1 and β-actin were as described above.

### Laser-assisted microdissection and subsequent RT-PCR

**Fig 1:**
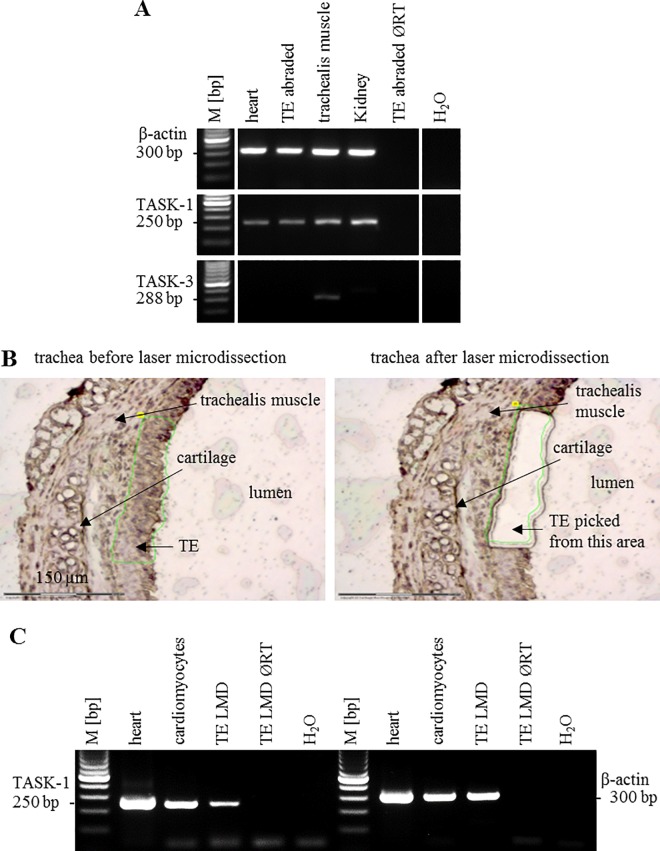
TASK-1 mRNA, but not TASK-3 mRNA, is expressed in the tracheal epithelium of mice. **(A)** RT-PCR from manually dissected tracheal epithelium (TE) using laboratory scissors, agarose gel. Irrelevant lanes from the gel were removed. Solid white lines indicate parts of the gel that were cut and pasted but from the same gel. **(B)** Laser-assisted microdissection of the tracheal epithelium. Area of the epithelium to be picked was first marked (left panel), the green lines show laser tracing. The marked piece of tissue (missing in right panel) was catapulted into the lid of a reaction tube and processed for RT-PCR. **(C)** RT-PCR from laser-microdissected (LMD) samples, agarose gel. β-Actin served as a housekeeping gene to control for PCR efficacy. Heart was used as a positive control for RT-PCR detection of TASK-1 mRNA. ØRT: control run without reverse transcriptase, H_2_O: control run without template, M: 100 base pair size marker.

Laser-assisted tissue microdissection was used to isolate cardiomyocytes and tracheal epithelium (n = 5 each) using a MicroBeam System (P.A.L.M. Microlaser Technologies, Bernried, Germany). Each dissected piece of tissue contained several cells (Fig 1B) and about 60 of such samples were collected for each specimen to be processed for RT-PCR. Cardiomyocytes were microdissected from the heart sections to serve as a positive control for laser microdissection, transcription, and amplification processes. Methods of cell picking, total RNA extraction, and cDNA synthesis followed by qualitative PCR were followed as described previously [25]. The sequences of primer sets for TASK-1 and β-actin used were as described above. The following control reactions were also included: cDNA of heart sample as positive control for PCR and negative controls were run without RT (ØRT) or without template (H_2_O). Resulted PCR products were analyzed on 2% ethidium bromide-stained agarose gels.

### Measuring PTS at the tracheal surface

Trachea preparation, imaging, and particle tracking were pursued as described previously [18]. First application of substances was done 45–55 min after tracheal dissection. The following substances were used in different experiments: Isoflurane (Abbott, Wiesbaden, Germany) dissolved in hydroxyethyl piperazine ethane sulphonic acid (HEPES)-Ringer solution (5.6 mM KCl, 1 mM MgCl_2_,10 mM HEPES, 136.4 mM NaCl, 11 mM glucose, 2.2 mM CaCl_2_, pH 7.4), Avertin® (Sigma-Aldrich, Steinheim, Germany) dissolved in water, anandamide (Sigma-Aldrich) dissolved in ethanol (Merck, Darmstadt, Germany), A293 [2-(butane-1-sulfonylamino)-N-[1-(R)-(6-methoxy-pyridin-3-yl)-propyl]-benzamide] gift from Sanofi Aventis, Frankfurt, Germany)] dissolved in dimethyl sulfoxide (DMSO, Sigma-Aldrich). ATP (Sigma-Aldrich) was administered at the end of each experiment to test the viability of the preparation. The total number of mice used in each experiment is indicated in the respective figure legends. To conduct the experiments where application of isoflurane was involved, an isoflurane vaporizer (Abbott) was used. The vaporizer was attached to a flask containing HEPES buffer. One millilitre of a 4% isoflurane solution was prepared and added to the incubation chamber containing 1 ml of HEPES buffer, resulting in a final isoflurane concentration of 2%.

### Statistical analysis

Statistical analysis was carried out using SPSS 11.5.1 software (SPSS Software, Munich, Germany). To select for the appropriate statistical test and data presentation, increases in PTS in response to the control test stimuli ATP and a cholinergic stimulus (n = 41) were pooled from this and parallel studies to achieve a sufficient number for analysis of the data distribution of the data set. The Kolmogorov-Smirnov test revealed no significant difference of the data set to normal distribution. Therefore, data are presented as mean ± standard error of the mean (SEM), and analysis was carried out with paired t-test and analysis of variance (ANOVA) with p values ≤ 0.05 and ≤ 0.01 considered as being significant and highly significant, respectively. P values presented in figures were obtained by t-test (two points from one experiment were compared) except those figures where values were obtained from ANOVA with post-hoc Bonferroni correction (same point from different experiments was compared).

## Results

### TASK-1 mRNA, but not TASK-3 mRNA, is detectable in the tracheal epithelium of mice

As our study was based on the presence of TASK-1 in the airways and its role in MCC, we performed PCR to evaluate the expression of TASK-1 mRNA in the tracheal epithelium. Since its function may be compensated by TASK-3 in TASK-1 deficiency [22], we also analyzed the tracheal epithelium for expression of TASK-3 mRNA. RT-PCR revealed bands of expected size for TASK-1 in the heart, tracheal epithelium, trachealis muscle, and kidney (Fig 1A). TASK-3 mRNA, however, was detectable in the trachealis muscle but not in the heart, tracheal epithelium, and kidney. Bands of equal intensities were obtained in all samples for β-actin which served as internal control.

Expression of TASK-1 mRNA by the tracheal epithelium was further validated by laser-assisted microdissection and subsequent RT-PCR (Fig 1B and 1C). TASK-1 transcripts were detected in the heart, cardiomyocytes, and, with lesser band intensity in agarose gels, in the tracheal epithelium. All samples exhibited prominent bands for β-actin which served as housekeeping gene control. In the course of data validation, we performed real-time RT-PCR using abraded tracheal epithelium from TASK-1 gene-deficient mice created by Aller et al. [22]. Unexpectedly, there was still 8% residual TASK-1 mRNA detectable in the epithelium of these TASK-1 KO mice. By qualitative PCR, no mRNA was detectable in the lung, cerebellum, and heart of the other strain of TASK-1 KO mice created by Mulkey et al. [23].

### Avertin®, but not isoflurane, reduces cilia-driven PTS at the mucosal surface of the mouse trachea

**Fig 2:**
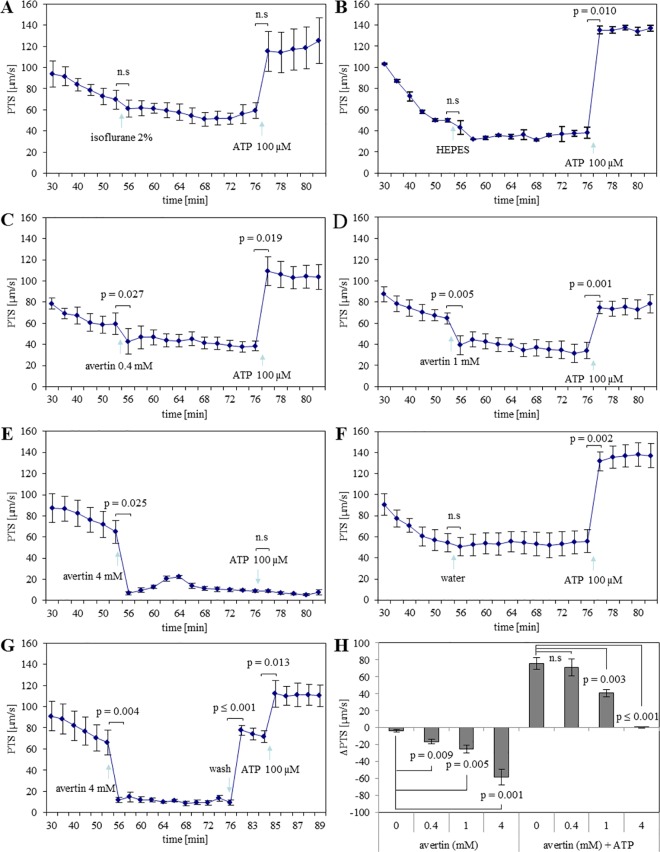
Anesthetics differentially decrease cilia-driven particle transport speed (PTS). **Isoflurane has no effect on PTS, whereas Avertin® reduces PTS. (A)** Isoflurane did not affect PTS. N = 3 tracheas from 3 animals. **(B)** HEPES (solvent of isoflurane) alone also had no effect on PTS. N = 2 tracheas from 2 animals. **(C)** Avertin® (0.4 mM) decreased PTS significantly. N = 3 tracheas from 3 animals. **(D)** Avertin® (1 mM) decreased PTS. N = 5 tracheas from 5 animals. **(E)** Avertin® (4 mM) robustly decreased basal PTS and ATP could not induce increase in PTS. N = 3 tracheas from 3 animals. **(F)** Water (solvent of Avertin®) had no effect on PTS. N = 4 tracheas from 4 animals. **(G)** Washing away of Avertin® from the chamber resulted in an increase in PTS, thus demonstrating viability of the trachea. N = 5 tracheas from 5 animals. P values ≤ 0.05 (paired t-test) are indicated. **(H)** Avertin® dose-dependently decreased basal PTS, and influenced ATP-stimulated PTS. The effects of different concentrations of Avertin® on PTS and their influence on ATP-stimulated PTS were compared with that of water after 1 min of their application. The y-axis shows the changes in PTS (ΔPTS) that occurred within 1 min after Avertin® application (left) and 1 min after ATP (100 μM) application in the continuous presence of Avertin® at different concentrations (right). Water was added instead of Avertin® in the solvent control, labelled here as “0 mM Avertin®”. P values ≤ 0.05 (ANOVA) are indicated. n.s: not significant.

To test if anesthetics target TASK-1 for modulating MCC in the trachea [4, 26], we employed two anesthetics, isoflurane and Avertin®. Against our expectations, 2% isoflurane did not affect basal PTS (Fig 2A), and ATP-stimulated increase in PTS also sustained in its presence. HEPES (solvent of isoflurane) also had no effect on PTS (Fig 2B). The effect of isoflurane was not significantly different from that of HEPES compared after 1 min of their application. In contrast to isoflurane, Avertin® dose-dependently decreased PTS with significant effects seen at all concentrations, i.e. 0.4 mM, 1 mM, and 4 mM (Fig 2C–2E). These effects were significantly different from the solvent (water) control (Fig 2H). The half maximal inhibitory concentration (IC50) of Avertin® was 1.2 mM.

Avertin® dose-dependently also diminished the stimulatory effect of subsequently administered ATP with a full blockade of ATP-induced increase in PTS in the presence of 4 mM Avertin® (Fig 2C–2F and 2H). This was not caused by a general toxic effect of Avertin® at 4 mM since the effect could be washed out by fresh medium (Fig 2G). As 1 mM Avertin® reduced basal as well as ATP-stimulated PTS, we used this concentration in the next experiments.

### Inhibition of TASK-1 channels does not affect cilia-driven PTS in WT mice

**Fig 3:**
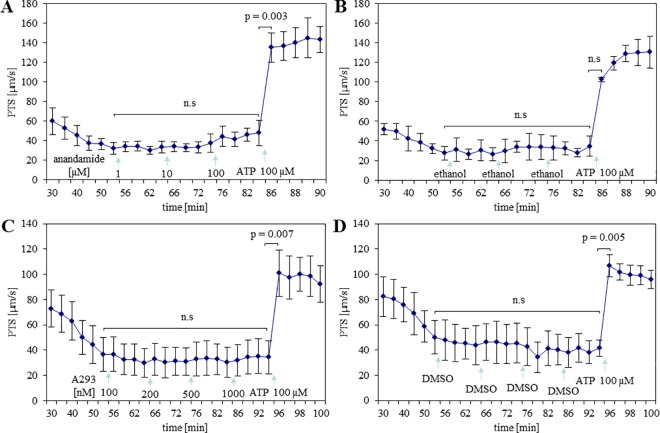
Inhibitors of TASK-1 channel have no effect on basal and subsequent ATP-stimulated particle transport speed (PTS). **(A)** Cumulative increase of anandamide concentration did not affect PTS. N = 3 tracheas from 3 animals. **(B)** Ethanol (solvent of anandamide) alone had no effect on PTS. N = 2 tracheas from 2 animals. **(C)** Cumulative increase of A293 concentration had no effect on PTS. N = 3 tracheas from 3 animals. **(D)** DMSO (solvent of A293) alone did not affect PTS. N = 3 tracheas from 3 animals (paired t-test). Effects of drugs were comparable to those of their solvents (ANOVA). n.s: not significant.

Anandamide at submicromolar concentration directly inhibits TASK-1 [27]. Against our expectations, anandamide at different concentrations neither increased PTS nor influenced reactivity to subsequent ATP stimulation in the concentration range of 1 μM to 100 μM (Fig 3A), similar to its solvent control (ethanol, Fig 3B). A293 at 200 nM concentration selectively inhibits TASK-1 channels as demonstrated in [21]. Similar to anandamide, cumulative application of 100 nM, 200 nM, 500 nM, and 1000 nM A293 did not increase PTS, and ATP-stimulated increase in PTS remained unaffected (Fig 3C). DMSO (solvent of A293) also did not influence these parameters (Fig 3D). Hundred micromolar anandamide and 1 μM A293 were used in the next experiments.

### Avertin®-induced decrease in PTS persists in WT mice in the presence of TASK-1 inhibitors and in TASK-1 KO mice

**Fig 4:**
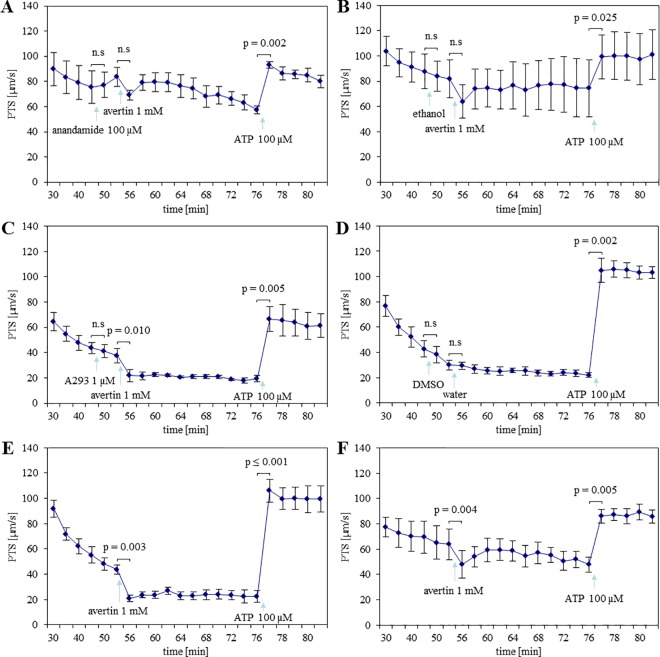
Avertin®-induced decrease in basal particle transport speed (PTS) remains unaffected in WT mice in the presence of TASK-1 inhibitors and in TASK-1 KO mice. **(A)** Avertin® in the presence of anandamide did not decrease PTS significantly. N = 4 tracheas from 4 animals. **(B)** Avertin® in the presence of ethanol (solvent of anandamide) also could not induce a decrease in PTS, so that the effect of Avertin® in the presence of anandamide (A) was not considered as a specific effect of TASK-1 inhibition. N = 4 tracheas from 4 animals. **(C)** Avertin® in the presence of A293 reduced PTS. N = 5 tracheas from 5 animals. **(D)** DMSO (solvent of A293) and water (solvent of Avertin®), both also had no effect on PTS. N = 5 tracheas from 5 animals. **(E, F)** Avertin®-induced decrease in basal PTS sustained in both strains of KO mice created by Aller et al. (E) and by Mulkey et al. (F). N = 4 tracheas from 4 animals. P values ≤ 0.05 (paired t-test) are indicated. n.s: not significant.

**Fig 5:**
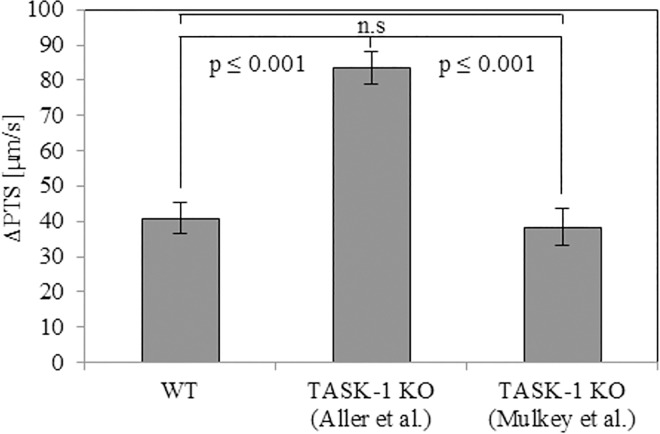
Avertin® does not influence subsequent ATP-stimulated increase in particle transport speed (PTS) in one strain of TASK-1 KO mice. ATP-stimulated increase in PTS (ΔPTS) measured after 1 min of ATP application was higher in TASK-1 KO mice created by Aller et al. as compared to that of KO mice created by Mulkey et al. and of WT mice. N = 4 and 5 tracheas from 4 KO animals each and 5 WT animals, respectively. P values ≤ 0.05 (ANOVA) are indicated. n.s: not significant.

To evaluate if Avertin® decreased PTS through targeting TASK-1 channels, we administered anandamide and A293 before applying Avertin®. In the presence of anandamide, Avertin® did not induce a significant decrease in PTS while the stimulatory effect of ATP was still observed (Fig 4A). This effect, however, was also seen when the solvent of anandamide, ethanol, was used alone, so that it was not considered as a specific effect of TASK-1 inhibition (Fig 4B). On the other hand, in the presence of A293, Avertin® still decreased PTS significantly, and ATP-induced increase in PTS remained unaffected (Fig 4C). Solvents of A293 and Avertin® in combination also did not influence PTS (Fig 4D). Next, we used two different strains of TASK-1 gene-deficient mice to elucidate if Avertin® reduced PTS through targeting/activating TASK-1 channels. In one strain of TASK-1 gene KO mice, created by Aller et al. [22], residual TASK-1 mRNA was detectable. Thus, to avoid doubts as to the validity of the data obtained with this strain, we decided to use another strain of KO mice created by Mulkey et al. [23] in which TASK-1 mRNA was not detectable. In both strains of TASK-1 KO mice, Avertin® significantly reduced basal PTS (Fig 4E and 4F). When we compared the effect of ATP on PTS after 1 min of its application, the ATP-stimulated increase in PTS was significantly higher in TASK-1 KO mice created by Aller et al. [22] as compared to that of WT mice and TASK-1 KO mice created by Mulkey et al. [23] (Fig 5). Since this effect was seen in only one of the KO mouse strains and was also not evident in experiments with the TASK-1 inhibitors anandamide and A293 (Fig 3A and 3C), we do not attribute it to the absence of TASK-1.

## Discussion

The major finding of this study is that, despite TASK-1 is expressed in the murine tracheal epithelium, neither inhibitors nor an activator of TASK-1 modulate cilia-driven particle transport at the mucosal surface. As for isoflurane, which had no impact on cilia-driven transport in our preparation, conflicting results have been reported previously. A decrease in CBF has been observed in the cultured rat tracheal epithelium [28], explanted murine trachea [29], and human nose [26, 30], whereas an increase was noted in the rabbit maxillary sinus in vivo [31]. Overall nasal MCC, measured in vivo by scintigraphy in mice, was not evidently altered in isoflurane anesthesia [4]. These largely variant findings may be ascribed to species differences or technical issues when working with a volatile anesthetic in aqueous solution, but differences in anatomical site of investigation also have to be taken into account, since basal CBF and its modulation can vary significantly along the airway tree [32–34].

On a molecular basis, differential expression of TASK-1 and TASK-3 and their species-specific properties might explain the range of effects evoked by isoflurane. These channels can form both heterodimers and homodimers, and their activity is differentially modulated by isoflurane, especially in rats. Isoflurane activates currents in rat TASK-3 and TASK-1/TASK-3 expressing cells while indeed inhibiting currents in solely rat TASK-1 expressing cells [20]. However, human TASK-1 expressed in *Xenopus* oocytes and murine TASK-1 expressed in human embryonic kidney 293 (HEK293) cells are activated by isoflurane [21, 35]. On this basis, the expected effect of isoflurane on the murine tracheal epithelium, in which our RT-PCR analysis revealed TASK-1 but no TASK-3 expression, shall be a hyperpolarization due to K^+^ efflux. Direct data on the link between membrane potential and CBF in airway cells are sparse and inconsistent. In isolated ciliated cells of the rabbit trachea, Ma and coworkers did not observe an impact of changes in membrane potential on CBF [36], whereas single ciliated cells of the mouse trachea, which matches more closely to the model in our study, responded to membrane hyperpolarization with enhanced Ca^2+^ influx through non-voltage-gated calcium channels with subsequent rise in CBF [37]. In this latter study, membrane hyperpolarization was achieved by diazoxide, an opener of ATP-sensitive K^+^ channels (K_ATP_). In contrast, we have not noted changes in cilia-driven PTS in our preparation utilizing isoflurane as activator of the two-pore domain K^+^ channel TASK-1. It is unclear, whether diazoxide and isoflurane influence membrane potential to a similar extent. Notably, it has been reported that rodent (rat) TASK-1 is much less sensitive to isoflurane than human TASK-1 [20], so that the possibility remains that it served as a comparatively weak stimulus. Still, the concentration we have used in the present study (2%, corresponding to 1.08 mM) is in the upper range of what has been used in electrophysiological studies on isolated and transfected cells (0.2–2 mM, mostly less than 1 mM; [5, 20, 35]), and half of that concentration (0.5 mM) had pronounced effects on currents in murine brainstem motoneurons [35]. Thus, albeit exact EC50 values are not available for isoflurane acting upon murine TASK-1, it shall be expected that our experimental setup would allow for efficient TASK-1 activation.

Hence, to further clarify whether modulation of TASK-1 open probability is linked to PTS in the mouse trachea, we investigated the effects of known inhibitors (anandamide, A293) and of genetic deletion of TASK-1 utilizing independently generated two strains of KO mice. Neither of these interventions, however, had an impact on PTS, despite both accelerating and decelerating effects could be evoked by ATP and Avertin®, respectively, demonstrating the suitability of the experimental setup to detect changes in either direction.

In neurons and carotid body glomus cells coexpressing TASK-1 and TASK-3, loss of one of these channels can be at least partly compensated by the other [35, 38]. For two reasons, this might have not masked effects in the present study utilizing only single TASK-1 KO mice. First, we have not observed TASK-3 expression in the tracheal epithelium, so that the data obtained from the single TASK-1 knockouts shall be conclusive. Second, anandamide and A293 address both TASK-1 and TASK-3, albeit not with the same affinities [21, 22], so that full inhibition also of potentially present TASK-3 homo- or heteromers should have been obtained.

Thus, the present data strongly argue against an involvement of TASK-1 in regulation of cilia-driven PTS in a mucus-free environment. This does not exclude, however, that volatile anesthetics such as isoflurane affect other parameters relevant for MCC via TASK-1. Viscosity of the mucus is an important factor in overall MCC, and this is strongly dependent on vectorial ion transport. Bupivacaine, an inhibitor of several two-pore domain K^+^ channels, including TASK-1 [39], inhibits both amiloride-sensitive Na^+^ absorption and forskolin-stimulated anion secretion in human bronchial epithelial cells [34], and chloride secretion in the rabbit trachea is also disturbed by halothane and isoflurane [40]. Since airway epithelial cells express multiple two-pore domain K^+^ channels [34], it remains to be determined which of them, if they do not contribute equally, is primarily responsible for this effect.

In contrast to isoflurane and TASK-1 inhibitors, Avertin® had a pronounced depressive effect on PTS at the tracheal surface. In line with this observation, Hua et al. observed a large reduction in nasal MCC in mice by pinhole gamma scintigraphy in response to Avertin® compared with isoflurane [4]. We are not aware of direct investigations of possible effects of Avertin® on TASK channels, e.g. by patch clamping of cells selectively expressing this channel. However, it is well suitable for anesthesia in TASK-1 KO mice [41] while these mice are less sensitive to the anesthetic effects of halothane and isoflurane [6]. This might indicate that the anesthetic effect of Avertin® is not mediated via TASK-1 channels. Clearly, its suppressive effect upon MCC is independent of TASK-1 since it persisted both in the presence of TASK-1 inhibitors and, most notably, in two different strains of TASK-1 KO mice.

In conclusion, TASK-1, a two-pore domain potassium channel being a molecular target of volatile anesthetics and here shown to be expressed by the murine respiratory epithelium, is not linked to modulation of cilia-driven particle transport in the murine trachea. An unrelated anesthetic, Avertin®, profoundly dampens PTS in a TASK-1-independent manner, showing that suppressive effects of anesthetics on MCC occur at multiple levels.
